# MDCK cells expressing constitutively active Yes-associated protein (YAP) undergo apical extrusion depending on neighboring cell status

**DOI:** 10.1038/srep28383

**Published:** 2016-06-21

**Authors:** Takanori Chiba, Erika Ishihara, Norio Miyamura, Rika Narumi, Mihoko Kajita, Yasuyuki Fujita, Akira Suzuki, Yoshihiro Ogawa, Hiroshi Nishina

**Affiliations:** 1 Department of Developmental and Regenerative Biology, Medical Research Institute, Tokyo Medical and Dental University (TMDU), 1-5-45 Yushima, Bunkyo-ku, Tokyo, Japan; 2 Department of Molecular Endocrinology and Metabolism, Graduate School of Medical and Dental Sciences, Tokyo Medical and Dental University (TMDU), 1-5-45 Yushima, Bunkyo-ku, Tokyo, Japan; 3 Division of Molecular Oncology, Institute for Genetic Medicine, Hokkaido University Graduate School of Chemical Sciences and Engineering, Kita 15, Nishi 7, Kita-ku, Sapporo, Hokkaido, Japan; 4 Division of Cancer Genetics, Medical Institute of Bioregulation, Kyushu University, 3-1-1 Maidashi Higashi-ku, Fukuoka, Japan; 5 Division of Molecular and Cellular Biology, Kobe University Graduate School of Medicine, 7-5-1 Kusunoki-cho, Chuo-ku, Kobe, Hyogo, Japan

## Abstract

Cell competition is a cell-cell interaction by which a cell compares its fitness to that of neighboring cells. The cell with the relatively lower fitness level is the “loser” and actively eliminated, while the cell with the relatively higher fitness level is the “winner” and survives. Recent studies have shown that cells with high Yes-associated protein (YAP) activity win cell competitions but the mechanism is unknown. Here, we report the unexpected finding that cells overexpressing constitutively active YAP undergo apical extrusion and are losers, rather than winners, in competitions with normal mammalian epithelial cells. Inhibitors of metabolism-related proteins such as phosphoinositide-3-kinase (PI3K), mammalian target of rapamycin (mTOR), or p70S6 kinase (p70S6K) suppressed this apical extrusion, as did knockdown of vimentin or filamin in neighboring cells. Interestingly, YAP-overexpressing cells switched from losers to winners when co-cultured with cells expressing K-Ras (G12V) or v-Src. Thus, the role of YAP in deciding cell competitions depends on metabolic factors and the status of neighboring cells.

Yes-associated protein (YAP) is a transcriptional co-activator that binds to transcription factors such as the TEA domain (TEAD) family to drive target gene expression^1^,^2^,^3^,^4^,^5^. YAP is negatively regulated by phosphorylation triggered by Hippo signaling. Phosphorylated YAP is retained in the cytoplasm by binding to phosphoserine/phosphothreonine-binding protein 14-3-3 and is subsequently degraded. Non-phosphorylated YAP is active and translocates to the nucleus where it exerts its co-activator function. Hippo-YAP signaling regulates organ size and cancer formation through effects on diverse cellular responses, including proliferation, contact inhibition and epithelial-mesenchymal transition.

During the cell-cell interaction termed “cell competition”, which was originally discovered in *Drosophila*^6^, a cell compares its fitness to that of its neighboring cells. Cells with a relatively higher fitness level become “winners” and survive, while cells with a relatively lower fitness level are “losers” and eliminated by either apoptosis or apical extrusion^7^,^8^,^9^,^10^,^11^,^12^,^13^. In *Drosophila*, *Minute* heterozygous cells have reduced ribosomal activity. When *Minute* heterozygous epithelial cells of *Drosophila* wing disc confront wild-type (WT) *Drosophila* cells, the *Minute* heterozygous cells are losers and killed by apoptosis^14^,^15^. Similarly, in mouse epiblasts or embryonic stem cells, cells with lower Myc levels are losers and undergo apoptosis^16^,^17^. In contrast, when Madin-Darby canine kidney (MDCK) epithelial cells expressing the oncogene proteins K-Ras (G12V) or v-Src are surrounded by non-transformed cells, the transformed MDCK cells are losers and removed by apical extrusion^18^,^19^.

Genetic screening for autosomal mutations that protect *Minute* heterozygous cells from death by cell competition identified mutations of Hippo signaling components as capable of suppressing the elimination of these cells^20^. Yorkie is the *Drosophila* homolog of YAP, and when Yorkie-overexpressing cells and WT cells coexist in *Drosophila*, the Yorkie-overexpressing cells are winners and the losing WT cells are eliminated by apoptosis^21^,^22^. In mammalian non-epithelial cells, when cells with relatively high TEAD activity are co-cultured with cells with relatively low TEAD activity, the former are winners and the latter are losers^23^. These results indicate that YAP is an evolutionarily conserved and critical determinant of cell competition, but the underlying molecular mechanisms are largely unknown.

In this study, we examine cell competition in a mammalian epithelial cell culture system and have made the unexpected discovery that cells overexpressing active YAP can become losers and undergo elimination by apical extrusion.

## Results

### Mammalian cells expressing constitutively active YAP undergo apical extrusion following contact with normal epithelial cells

**Figure 1:**
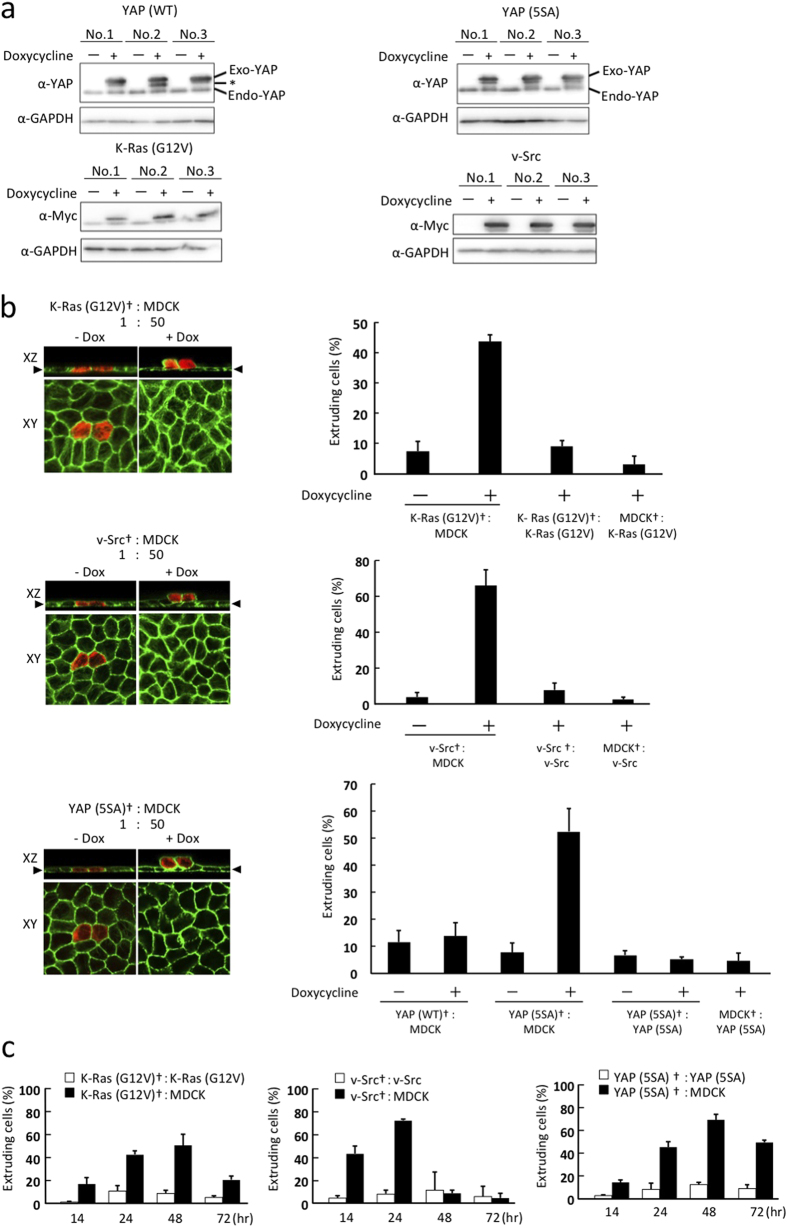
YAP (5SA)-expressing cells undergo apical extrusion when surrounded by normal epithelial cells. (**a**) Immunoblots to detect (top) the endogenous (Endo) and exogenous (Exo) forms of YAP, or (bottom) Myc-tagged K-Ras (G12V) or v-Src, in three lines each of MDCK cells expressing YAP (WT), YAP (5SA), K-Ras (G12V) or v-Src in a Dox-dependent manner. GAPDH, loading control. Results are representative of 3 trials. * Indicates a degradation product of Exo-YAP. (**b**) Left panels: Confocal images of xz and xy sections of plates in which labeled (†) K-Ras (G12V), v-Src or YAP (5SA) cells (red) were co-cultured 1:50 with non-labeled normal MDCK cells. Cells were fixed after 24 hr incubation with (+) or without (−) Dox and stained with phalloidin (green). Arrowheads point to xy plane of cells. Right panels: Quantitation of the percentage of extruding labeled cells among a 1:50 mixture of the indicated co-cultured cell lines after 24 hr with/without Dox. Data are the mean ± s.d. (n = 3/group) of three independent experiments. (**c**) Time course of the apical extrusion of labeled cells among 1:50 mixtures of the indicated labeled and non-labeled cell lines. The percentage of extruding cells in each case was examined at the indicated times after Dox addition. Data are the mean ± s.d. (n = 3/group) of three independent experiments.

To examine the role of YAP in cell competition, we used established mammalian epithelial cell culture systems^18^,^19^ to evaluate the fate of YAP-overexpressing MDCK cells cultured in a monolayer with normal MDCK cells. Firstly, we established MDCK cell lines that expressed WT YAP [YAP (WT)], or a constitutively active form of YAP [YAP (5SA)] in which the five Ser residues phosphorylated by Hippo signaling can be replaced with Ala in a doxycycline (Dox)-dependent manner (Fig. 1a). There were no significant differences in cell proliferation or survival between normal MDCK cells and MDCK cells expressing YAP (WT) or YAP (5SA) (Supplementary Fig. 1). To track the consequences of cell competition, we labeled YAP (WT)- or YAP (5SA)-expressing MDCK cells with a red fluorescent dye (CMTPX) and mixed them with normal MDCK cells at ratio of 1:50 (mosaic condition). As a positive control, we also labeled previously established MDCK cell lines expressing K-Ras (G12V) or v-Src in a Dox-dependent manner^18^,^19^. Reciprocally, we labeled normal MDCK cells with CMTPX and mixed them with the above overexpressing cells at ratio of 1:50. These cell mixtures were cultured on a collagen matrix in the absence of Dox until a monolayer was formed. YAP (WT), YAP (5SA), K-Ras (G12V) or v-Src expression was then induced by adding Dox for 24 hr. The fate of the labeled cells was determined by fixing them and examining them by confocal microscopy.

We observed that the percentage of labeled K-Ras (G12V)- or v-Src-expressing MDCK cells [but not YAP (WT)-expressing MDCK cells] that underwent apical extrusion following Dox induction increased significantly (from 10% to 40% or 60%, respectively) when these cells were surrounded by normal cells (Fig. 1b). Surprisingly, the percentage of labeled YAP (5SA) cells that underwent Dox-dependent apical extrusion when in contact with unlabeled normal cells also increased from 10% to 50%. On the other hand, the percentage of apically-extruded labeled normal MDCK cells did not rise when these cells were flanked by either K-Ras (G12V), v-Src or YAP (5SA) cells. When we investigated the time course of these events, we found that labeled K-Ras (G12V), v-Src and YAP (5SA) cells surrounded by normal cells underwent apical extrusion between 14–24 hr after Dox addition (Fig. 1c). These results indicate that, in contrast to the case in *Drosophila*, mammalian cells expressing constitutively active YAP lose cell competitions with normal cells and undergo apical extrusion.

### The TEAD binding domain and PDZ binding motif of YAP are important for the apical extrusion of YAP (5SA) cells

**Figure 2:**
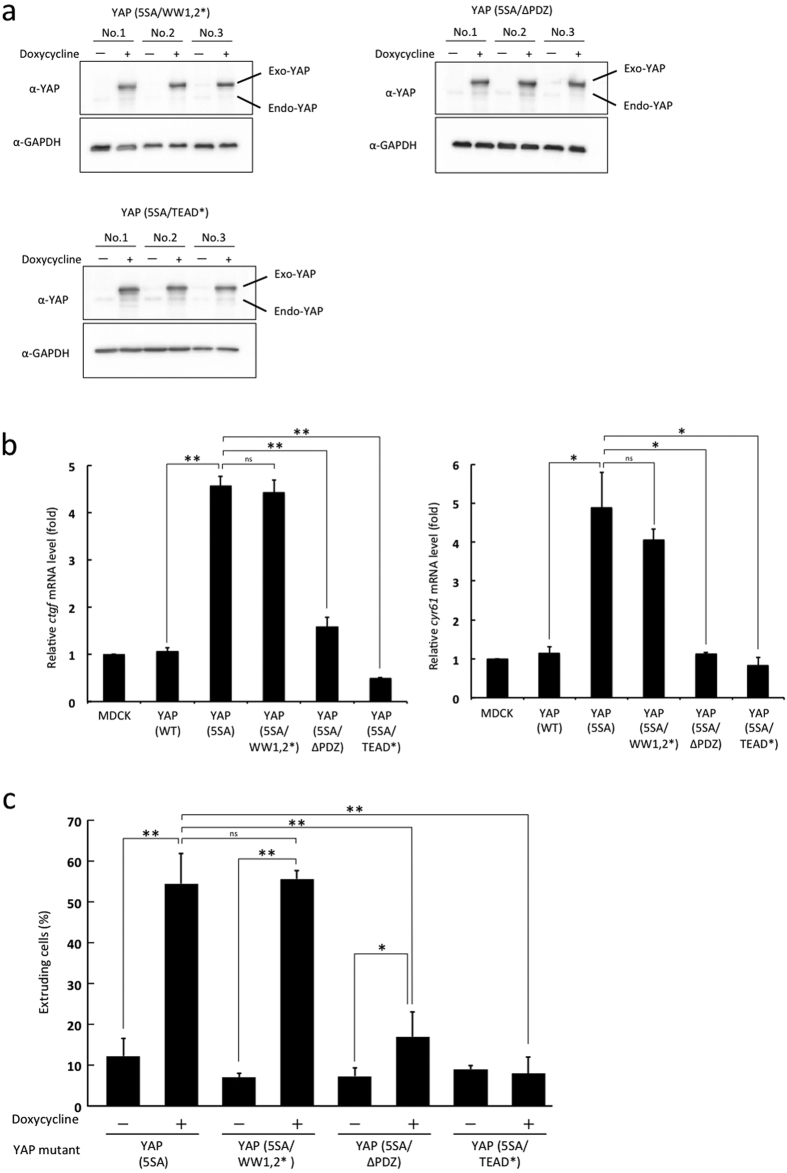
Identification of YAP domains required for cell extrusion. (**a**) Immunoblots to detect the indicated YAP isoforms in the indicated modified YAP (5SA) cell lines with/without Dox. *, mutation. Δ, deletion. Data were analyzed as in Fig. 1a. (**b**) Quantitation of RT-PCR analysis to detect *ctgf* and *cyr61* mRNAs in monocultures of the indicated cell lines. Total RNA was extracted 24 hr after Dox addition. Data were normalized to *gapdh* mRNA and expressed relative to the value of the normal MDCK sample (set to 1). (**c**) Quantitation of the percentage of apically extruded cells of the indicated mutant cell lines among co-cultures of labeled YAP mutant-expressing MDCK cells mixed 1:50 with non-labeled normal MDCK cells. Cells were fixed after 24 hr incubation with/without Dox. Data are the mean ± s.d. (n = 3/group) of three independent experiments. ns, not significant, *P < 0.05, **P < 0.01.

The mammalian YAP protein contains the TEAD binding domain, 14-3-3 binding domain, WW1 and WW2 domains, SH3-binding motif, transcriptional activation domain and PDZ binding motif^24^. To determine which of these domains were required for the apical extrusion of YAP (5SA) cells, we used site-directed mutagenesis in YAP (5SA) cells to introduce mutations (^*^) or deletion (Δ) into these domains and established MDCK cell lines expressing YAP (5SA/TEAD*), YAP (5SA/WW1,2*) or YAP (5SA/ΔPDZ) in a Dox-dependent manner (Fig. 2a). We evaluated the transcriptional co-activator activity of these mutated YAP proteins by measuring mRNA levels of the YAP-TEAD target genes *connective tissue growth factor* (*ctgf*) and *cysteine-rich angiogenic inducer 61* (*cyr61*) in each YAP (5SA) mutant cell line (Fig. 2b). Compared to those in YAP (WT) cells, the expression levels of *ctgf* and *cyr61* mRNAs were markedly raised in YAP (5SA) and YAP (5SA/WW1,2*) cells and slightly elevated in YAP (5SA/ΔPDZ) cells, but not increased in YAP (5SA/TEAD*) cells. Next, we examined the effect of the mutated YAP domains on apical extrusion induced by co-culture with normal cells. The percentage of extruded YAP (5SA/WW1,2*) cells was almost the same as that of YAP (5SA) cells cultured under these conditions, while that of YAP (5SA/ΔPDZ) cells was significantly reduced and that of YAP (5SA/TEAD*) cells was completely suppressed (Fig. 2c). These data indicate that the expression of TEAD-dependent genes and the presence of YAP’s PDZ binding motif are important for the apical extrusion of YAP (5SA) cells.

### Effects of chemical inhibitors on the apical extrusion of YAP (5SA) cells

**Figure 3:**
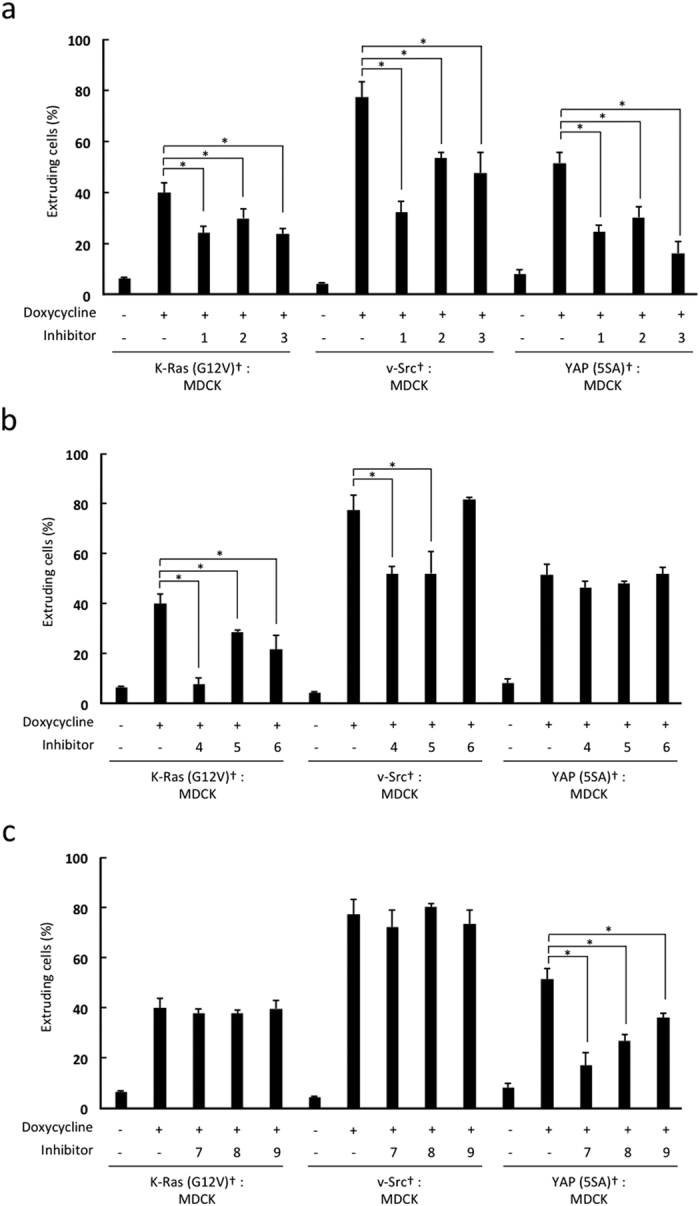
Effect of chemical inhibitors on apical extrusion. (**a–c**) Quantitation of percentages of apically extruded cells in co-cultures of labeled (†) K-Ras (G12V), v-Src or YAP (5SA) cells that were mixed 1:50 with non-labeled normal MDCK cells and incubated for 24 hr with Dox plus various inhibitors as follows: 1, cytochalasin D; 2, bisindolylmaleimide I; 3, withaferin A; 4, U0126; 5, (S)-(-)-blebbistatin; 6, JTE-013; 7, LY294002; 8, rapamycin; and 9, PF-4708671. (**a**) Inhibitors that suppressed apical extrusion of K-Ras (G12V), v-Src and YAP (5SA) cells; (**b**) inhibitors that suppressed apical extrusion of K-Ras (G12V) and/or v-Src but not YAP (5SA) cells; and (**c**) inhibitors that suppressed apical extrusion of YAP (5SA) cells only. Control cells were mixtures incubated for 24 hr with/without Dox in the absence of any inhibitor. Data are the mean ± s.d. of three independent experiments. *P < 0.01.

To elucidate the molecular mechanisms of apical extrusion of YAP (5SA) cells, we examined the effects of chemical inhibitors. Our results allowed us to separate these inhibitors into three classes (Fig. 3). The first class of inhibitors suppressed the apical extrusion of K-Ras (G12V), v-Src and YAP (5SA) cells and included cytochalasin D, an inhibitor of actin polymerization; bisindolylmaleimide I, an inhibitor of protein kinase C (PKC); and withaferin A, an inhibitor of vimentin (Fig. 3a). The second class of inhibitors suppressed apical extrusion of K-Ras (G12V) and/or v-Src cells but not YAP (5SA) cells and included blebbistatin, an inhibitor of myosin-II; U0126, an inhibitor of MEK; and JTE-013, a sphingosine-1-phosphate receptor-2 antagonist (Fig. 3b). The third class of inhibitors suppressed the apical extrusion of YAP (5SA) cells only and included LY294002, an inhibitor of phosphoinositide-3-kinase (PI3K); rapamycin, an inhibitor of mammalian target of rapamycin (mTOR); and PF-4708671, an inhibitor of p70S6 kinase (p70S6K) (Fig. 3c). Thus, apical extrusion of these overexpressing cells involves both shared and specific factors, depending on the gene mutation.

### Vimentin and filamin in neighboring normal MDCK cells promote the apical extrusion of YAP (5SA) cells

**Figure 4:**
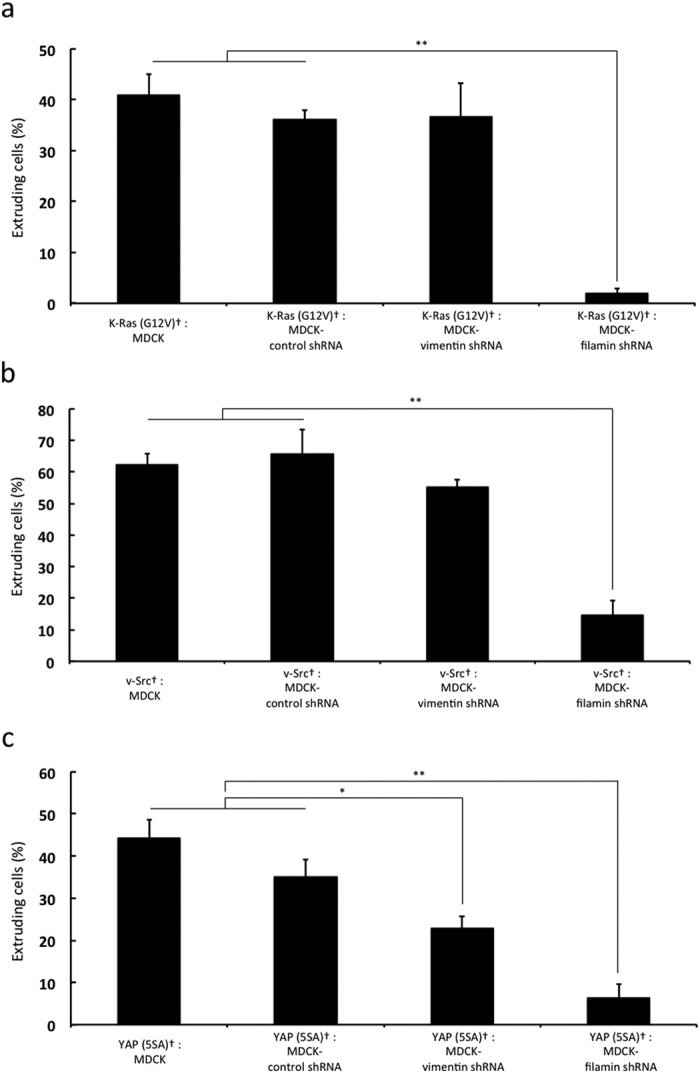
Effects on apical extrusion of knockdown of vimentin or filamin in neighboring cells. (**a–c**) Quantitation of percentages of apically extruded cells in co-cultures of labeled (†) (**a**) K-Ras (G12V), (**b**) v-Src or (**c**) YAP (5SA) cells mixed 1:50 with non-labeled normal MDCK cells, or MDCK cells expressing control shRNA or vimentin shRNA or filamin shRNA, as indicated. To induce sufficient knockdown of vimentin and filamin protein, cells expressing vimentin shRNA or filamin shRNA were incubated with Dox for 72 or 48 hr, respectively, before induction of K-Ras (G12V), v-Src or YAP (5SA). Data are the mean ± s.d. of three independent experiments. ns, not significant. *P < 0.05, **P < 0.01.

It has been previously reported that the presence in normal cells of vimentin, an intermediate filament protein, and filamin, a homodimeric actin-binding protein, is critical for inducing the apical extrusion of neighboring transformed cells^25^. Our data in Fig. 3a implicated vimentin and PKC in the apical extrusion of YAP (5SA) cells, and PKC is known to be involved in filamin-mediated vimentin phosphorylation^26^. We therefore used MDCK cells expressing Dox-inducible vimentin shRNA or filamin shRNA to determine if vimentin and filamin in neighboring normal cells were required for the apical extrusion of YAP (5SA) cells. The percentage of apically extruded K-Ras (G12V) cells was reduced when these cells were flanked by filamin-depleted normal MDCK cells (Fig. 4a), whereas the percentage of apically extruded v-Src cells was decreased when neighboring normal MDCK cells were depleted of either vimentin or filamin (Fig. 4b). Interestingly, the percentage of apically extruded YAP (5SA) cells was also suppressed by the presence of surrounding vimentin plus filamin-knockdown MDCK cells (Fig. 4c). These data suggest that vimentin and filamin in neighboring normal cells play important roles in deciding mammalian cell competitions regardless of YAP activity.

### Different lines of neighboring cells have differential effects on the apical extrusion of YAP (5SA) cells

**Figure 5:**
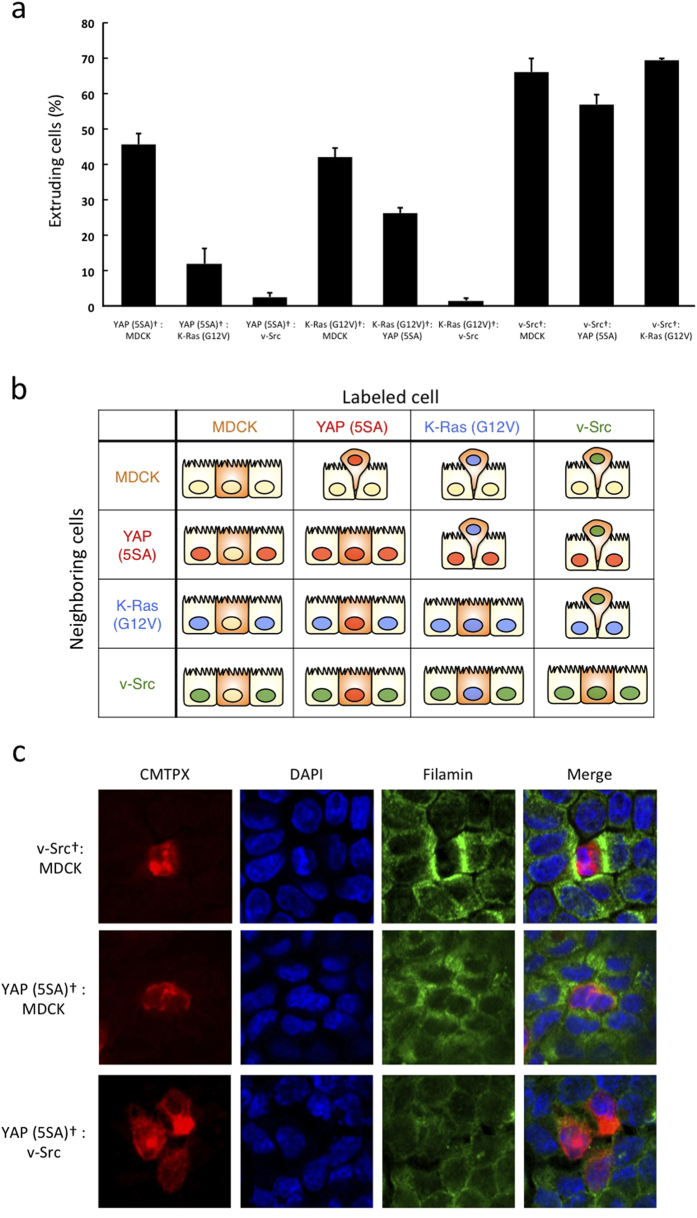
Comparison of the strength of apical extrusion-inducing activities of K-Ras (G12V), v-Src, YAP (5SA) and normal MDCK cells. (**a**) Quantitation of percentage of apically extruded cells when the indicated labeled (†) K-Ras (G12V), v-Src or YAP (5SA) cells were co-cultured 1:50 with non-labeled K-Ras (G12V), v-Src, YAP (5SA) or normal MDCK cells, as indicated. Cells were incubated for 24 hr with Dox prior to fixation. Data are the mean ± s.d. of three independent experiments. (**b**) Schematic table illustrating the results in (**a**). (**c**) Immunofluorescent staining of CMTPX (red), DAPI (blue), and filamin (green) in (top) labeled v-Src cells that were co-cultured 1:50 with non-labeled normal MDCK cells; (middle) labeled YAP (5SA) cells co-cultured 1:50 with non-labeled normal MDCK cells; and (bottom) labeled YAP (5SA) cells co-cultured 1:50 with non-labeled v-Src cells.

To compare the strengths of apical extrusion-inducing activity among K-Ras (G12V)-, v-Src-, YAP (5SA)-overexpressing and normal MDCK cells, we investigated the fate of overexpressing cells surrounded by various lines of overexpressing cells or normal MDCK cells. When YAP (5SA) cells were co-cultured with K-Ras (G12V) or v-Src cells, the percentage of apically extruded YAP (5SA) cells was profoundly decreased compared to that observed in co-cultures with normal MDCK cells (Fig. 5a). Similarly, when K-Ras (G12V) cells were flanked by v-Src cells, the apical extrusion of K-Ras (G12V) cells was completely suppressed. In contrast, when v-Src cells were surrounded by YAP (5SA) or K-Ras (G12V) cells, the percentage of apically extruded v-Src cells did not decrease. In summary, YAP (5SA) cells were better at inducing the apical extrusion of surrounding cells than K-Ras (G12V) and v-Src cells, but weaker than normal MDCK cells [normal MDCK > YAP (5SA) > K-Ras (G12V) > v-Src cells; Fig. 5b]. These results indicate that YAP (5SA) cells can switch from loser to winner status in cell competition depending on the status of the neighboring cells.

Our results in Fig. 4 indicated that filamin in neighboring normal cells plays a crucial role in inducing apical extrusion. Previous reports have shown that filamin accumulation in neighboring cells surrounding v-Src cells influences apical extrusion-inducing activity^25^. With this observation in mind, we used anti-filamin immunostaining to examine filamin levels in normal MDCK cells surrounding MDCK cells expressing v-Src or YAP (5SA). We found that filamin did indeed accumulate in neighboring normal cells surrounding v-Src cells (Fig. 5c). Interestingly, a lower level of filamin accumulation was observed in neighboring normal cells surrounding YAP (5SA) cells that underwent apical extrusion. In contrast, no filamin accumulation was observed in v-Src cells surrounding YAP (5SA) cells that did not undergo apical extrusion. These results indicate that filamin accumulation in neighboring cells correlates with the apical extrusion-inducing activity of these cells.

## Discussion

In this study, we found that mammalian cells expressing constitutively active YAP that were co-cultured with normal cells were losers in cell competition and underwent apical extrusion. We showed that the expression of TEAD-dependent genes, as well as that of several other molecules, including those involved in mTOR signaling, was required for this apical extrusion. Surprisingly, we found that YAP (5SA) cells could become either losers or winners of cell competitions depending on the status of neighboring cells.

As illustrated in Supplementary Fig. 2a and consistent with previous reports^27^,^28^, YAP (WT) shuttles between the cytoplasm and nucleus even when it is phosphorylated. YAP’s PDZ binding motif is necessary for YAP nuclear localization due to its interaction with the tight junction protein zonula occludens 2 (ZO-2)^29^. Accordingly, in previous work, we demonstrated that YAP (5SA/ΔPDZ) mutant protein is mainly located in the cytoplasm^30^. In this study, we showed that the percentage of YAP (5SA/ΔPDZ) cells that underwent apical extrusion when surrounded by normal cells was profoundly suppressed compared with that of YAP (5SA) cells, but slightly increased compared with YAP (WT) cells (Fig. 2). These results can be explained by YAP (5SA)’s altered cellular localization and TEAD-dependent gene expression.

Our data also show that PI3K, mTOR and p70S6K play important roles in the apical extrusion of YAP (5SA) cells (Fig. 3). Based on our results and previous reports^25^,^31^, we have developed a model for these functions that is illustrated in Supplementary Fig. 2b. PI3K, mTOR and p70S6K are involved in metabolic processes such as lipogenesis, protein synthesis and glycolysis^32^,^33^. In *Drosophila*, the *Minute* gene, which encodes a ribosomal protein, influences cell competition, suggesting the importance of protein synthesis for this event^14^,^15^. In addition, altered glucose metabolism is reportedly crucial for *Drosophila* cell competition induced by Myc overexpression^34^. When Myc-expressing *Drosophila* cells confront WT cells, the Myc-expressing cells enhance their glycolytic flux and increase their fitness and proliferation, garnering them winner status. These results are consistent with our findings and suggest that the metabolic changes occurring in YAP (5SA) cells are required for their apical extrusion.

When normal mammalian cells are co-cultured with transformed cells, filamin-mediated regulation of the dynamic movements of vimentin in the normal cells generates contractile forces that promote the apical extrusion of the transformed cell from the epithelial monolayer^25^, a result we confirmed (Fig. 4). We also showed that YAP (5SA) cells did not extrude apically when flanked by K-Ras (G12V) or v-Src cells (Fig. 5a). These results can be explained if expression of K-Ras (G12V) or v-Src downregulates the activity of filamin and/or vimentin in neighboring cells, or if expression of K-Ras (G12V) or v-Src in neighboring cells suppresses YAP-induced signaling pathways in YAP (5SA) cells. These alternatives are under investigation.

In *Drosophila,* Yorkie-overexpressing epithelial cells become winners in cell competition^21^,^22^, and “winning” mammalian non-epithelial cells reportedly show higher TEAD activity^23^. However, our study showed that, in co-cultures of mammalian epithelial cells, YAP (5SA) cells were losers and underwent apical extrusion when surrounded by normal cells (Fig. 1). On the other hand, these same YAP (5SA) cells became winners when they were co-cultured with K-Ras (G12V) or v-Src cells (Fig. 5). These results indicate that whether a cell becomes the winner or loser in a cell competition depends on the species, cell type and microenvironment. In other words, winner or loser status is not absolute or simply determined during cell competition but instead is a relative attribute governed by a complex set of circumstances.

## Methods

### Antibodies and inhibitors

Mouse anti-Myc (sc-40) and rabbit anti-YAP (sc-10547) antibodies (Abs) were purchased from Santa Cruz, mouse anti-GAPDH (CA92590) Ab was from Merck Millipore, and mouse anti-filamin (F6682) Ab was from Sigma-Aldrich. For immunoblotting, Abs were used at 1:3000 (anti-Myc), 1:1000 (anti-YAP) and 1:5000 (anti-GAPDH) dilution, respectively. For immunofluorescence, anti-filamin Ab was used at 1:100 dilution. The chemical inhibitors cytochalasin D (0.4 μM), JTE-013 (10 μM) and PF-4708671 (10 μM) were purchased from Sigma-Aldrich; bisindolylmaleimide I (10 μM), LY294002 (10 μM) and rapamycin (0.1 μM) from Calbiochem; withaferin A (2.5 μM) from Santa Cruz; U0126 (10 μM) from Promega; and (S)-(-)-blebbistatin (30 μM) from Toronto Research Chemicals. Inhibitors and Dox were purchased from Apollo Scientific and were added simultaneously to cultures.

### Cell lines and culture

The full-length human YAP cDNA was amplified by polymerase chain reaction (PCR) and ligated to XbaI restriction sites of the expression vectors used. The YAP (5SA), YAP (5SA/TEAD*), YAP (5SA/WW1,2*) and YAP (5SA/ΔPDZ) mutants were described in a previous study^30^. Constructs encoding YAPs, K-Ras (G12V) and v-Src were cloned into the pEN-TRE vector from Addgene. These vectors and pSLIK-neo were recombined using LR Clonase enzyme from Gateway. For the generation of lentivirus preparations, subconfluent 293T packaging cells on a 10 cm plate were transfected with pSLIK-Neo-cDNA construct, pRSV-Rev, pMDLg/pRRE and pMD2.G (pVSV) vectors using Lipofectamine 2000 from Invitrogen. After 48 hr, supernatants containing lentivirus were collected for infection of MDCK cells. To establish MDCK cells expressing YAPs, K-Ras (G12V) or v-Src in a Dox-dependent manner, one infected cell was seeded in each well of a 96 well plate and cells were cultured for three weeks in the presence of neomycin (G418) to select for resistance. Expression of YAPs, K-Ras (G12V) or v-Src cDNAs was induced by the addition of 2 μg/ml Dox to the culture medium. MDCK cells stably expressing control shRNA (Luciferase shRNA: 5′-GATCCCCTGAAACGATATGGGCTGAATTCAAGAGATTCAGCCCATATCGTTTCATTTTTC-3′), filamin shRNA (5′-GATCCCCGCTGGAGTGCCAGCTGAATTTCAAGAGAATTCAGCTGGCACTCCAGCTTTTTC-3′) or vimentin shRNA (5′-GATCCCCGCTGCTAACTACCAAGACATTCAAGAGATGTCTTGGTAGTTAGCAGCTTTTTC-3′) in a Dox-dependent manner were described previously^25^. All MDCK cell lines were maintained in Dulbecco’s modified Eagle’s medium (DMEM) supplemented with 10% fetal bovine serum.

### Immunoblotting

Immunoblotting was performed as described previously^35^. Blots were incubated overnight at 4 °C with anti-Myc, anti-YAP or anti-GAPDH Abs. Primary Abs were detected by incubation with anti-mouse or anti-rabbit peroxidase-conjugated secondary Abs from Santa Cruz as previously described^35^. Proteins were visualized using the SuperSignal West Femto Kit (Pierce) and a ChemiDoc XRS system (Bio-Rad).

### Quantitative real-time RT-PCR

Quantitative real-time RT-PCR was performed as described previously^36^. Primers used for RT-PCR analysis of mRNA expression were as follows: for *ctgf*, 5′-CTTGTGAAGCTGACCTGGAAG-3′ and 5′-CACAGAACTTAGCCCGGTATG-3′; for *cyr61*, 5′-GGCTGGAATGCAATTTCG-3′ and 5′-TCCCCATTCTGGTAGATTCG-3′; and for *gapdh*, 5′-ACGGCACAGTCAAGGCTGAG-3′ and 5′-CAGCATCACCCCATTTGATGTTGG-3′.

### Apical extrusion assay

Apical extrusion in collagen was evaluated as previously described^18^. Briefly, type I collagen was obtained from Nitta Gelatin (Nitta Cellmatrix type 1-A) and neutralized on ice to a final concentration of 2 mg/ml according to the manufacturer’s instructions. Glass coverslips in 35 mm glass-bottom dishes were coated with 200 μl of neutralized collagen and allowed to solidify for 30 min at 37 °C. Cells were plated at 1.5–2 × 10^6^ per well onto the collagen matrix. MDCK cells expressing YAPs, K-Ras (G12V) or v-Src were mixed with normal MDCK cells at a ratio of 1:50. After incubation for 16–24 hr at 37 °C, 2 μg/ml Dox was added to induce cDNA expression. After incubation for 24 hr, Dox-treated cells on collagen were fixed with 4% paraformaldehyde/PBS for 10 min at 37 °C, washed twice in PBS, and incubated with 0.1% Triton X-100/PBS for 10 min. Cells were then incubated with Hoechst stain and phalloidin/PBS for 1 hr to visualize extrusion.

To quantify the percentage of extruding cells, normal MDCK cells and MDCK cells expressing YAPs, K-Ras (G12V) or v-Src were labeled with CMTPX from Invitrogen according to the manufacturer’s instructions. Confocal microscopy was used to count the number of labeled extruding cells and the number of labeled non-extruding cells in the same culture. The percentage of extruding cells was calculated as the number of labeled extruding cells over this number plus the number of labeled non-extruding cells x 100%. At least 100 labeled cells were counted per culture.

### Immunofluorescence

Micro cover glasses (MATSUNAMI, 18 × 18 mm, 0.12–0.17 mm) were placed in 35 mm dishes and coated with 1 ml of collagen matrices. Cell mixtures were cultured on these collagen matrices for 24 hr at 37 °C until a monolayer was formed. Dox (2 μg/ml) was added for 18 hr [mixed culture of normal MDCK plus v-Src cells or v-Src plus YAP (5SA) cells] or 21 hr [mixed cultures of normal MDCK plus YAP (5SA) cells]. Cells were washed three times in cold PBS and fixed in methanol for 2.5 min at −20 °C. Fixed cells were blocked for 1 hr in 1% BSA/PBS, and incubated with primary Abs for 16 hr at 4 °C, and then incubated with Alexa-488-conjugated secondary Abs for 1hr at room temperature. Immunostained cells were incubated with Hoechst dye in 1% BSA/PBS for 15 min and mounted with Mowiol on cover glasses (MATSUNAMI, 24 × 60 mm, 0.12–0.17 mm). Immunofluorescent images were captured and analyzed using a LSM710 Zeiss confocal microscope.

### Statistics

Two-tailed Student’s t-tests were used to determine p values. P values of 0.05 were considered statistically significant.

## Additional Information

**How to cite this article**: Chiba, T. *et al*. MDCK cells expressing constitutively active Yes-associated protein (YAP) undergo apical extrusion depending on neighboring cell status. *Sci. Rep.* **6**, 28383; doi: 10.1038/srep28383 (2016).

## Supplementary Material

Supplementary Information
