# Supplementary material for: MDCK cells expressing constitutively active Yes-associated protein (YAP) undergo apical extrusion depending on neighboring cell status
Source: Sci Rep. 2016 Jun 21;6:28383. doi: 10.1038/srep28383 (PMC4914932; doi:10.1038/srep28383)
Supplement: Supplementary Information [file srep28383-s1.pdf]

Supplementary Information

MDCK cells expressing constitutively active Yes-associated protein (YAP) undergo apical extrusion depending on neighboring cell status

Takanori Chiba[1,2,*], Erika Ishihara[1,*], Norio Miyamura[1], Rika Narumi[3], Mihoko Kajita[3], Yasuyuki Fujita[3], Akira Suzuki[4,5], Yoshihiro Ogawa[2] and Hiroshi Nishina[1]

[1]Department of Developmental and Regenerative Biology, Medical Research Institute, Tokyo Medical and Dental University (TMDU), 1-5-45 Yushima, Bunkyo-ku, Tokyo, Japan

[2]Department of Molecular Endocrinology and Metabolism, Graduate School of Medical and Dental Sciences, Tokyo Medical and Dental University (TMDU), 1-5-45 Yushima, Bunkyo-ku ,Tokyo, Japan

[3]Division of Molecular Oncology, Institute for Genetic Medicine, Hokkaido University Graduate School of Chemical Sciences and Engineering, Kita 15, Nishi 7, Kita-ku, Sapporo, Hokkaido, Japan

[4]Division of Cancer Genetics, Medical Institute of Bioregulation, Kyushu University, 3-1-1 Maidashi Higashi-ku, Fukuoka, Japan

[5]Division of Molecular and Cellular Biology, Kobe University Graduate School of Medicine, 7-5-1 Kusunoki-cho, Chuo-ku, Kobe, Hyogo, Japan

a

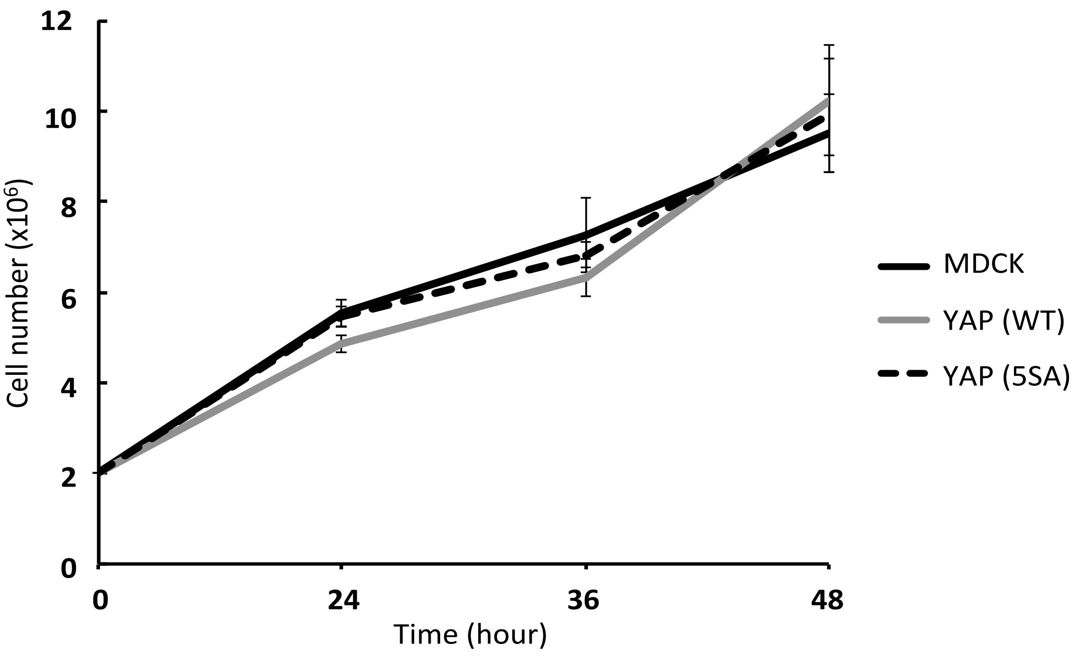

12
10
8
6
4
2
0
Cell number (x10^6)
0
24
36
48
Time (hour)
MDCK
YAP (WT)
YAP (5SA)

b

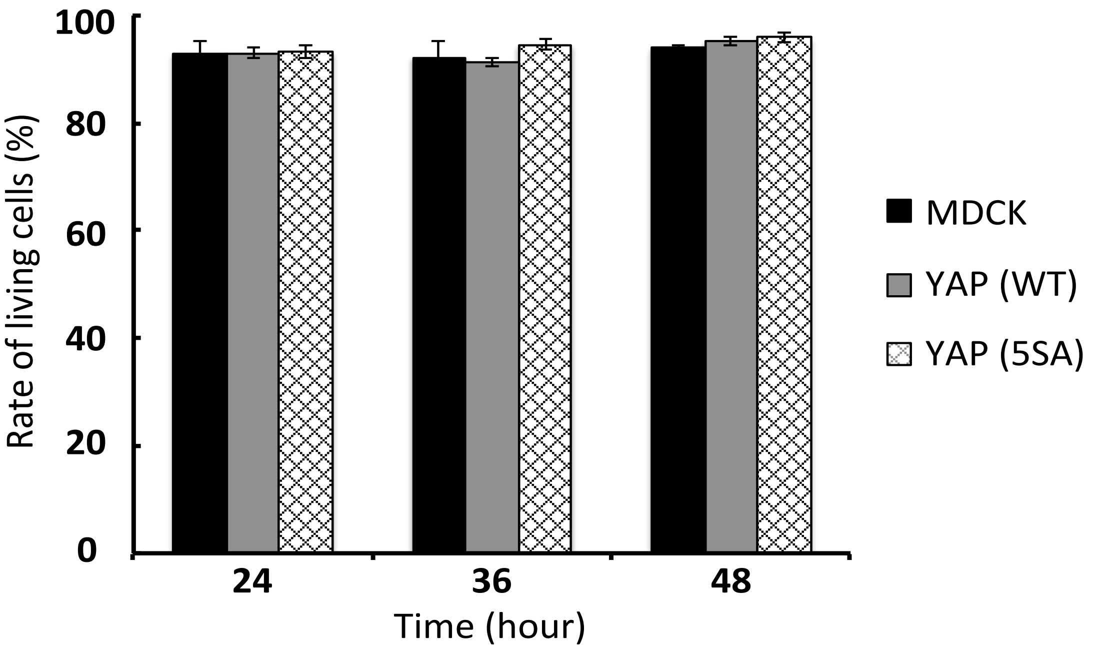

100
80
60
40
20
0
Rate of living cells (%)
24
36
48
Time (hour)
MDCK
YAP (WT)
YAP (5SA)

Supplementary Figure 1. Comparison of cell numbers and apoptosis in normal MDCK cells and Dox-treated MDCK cells expressing YAP (WT) or YAP (5SA).

(a) Cells ($2x10^6$) were seeded into 35 mm dishes and Dox was added after 24 hr. Quantitation of cell numbers of normal MDCK cells, and MDCK cells expressing YAP (WT) or YAP (5SA), was performed at 24, 36 and 48 hr after seeding. (b) Percent cell viability in the cultures in (a) was evaluated using trypan blue (T8154, Sigma-Aldrich). Data are the mean ± s.d. (n=3) of three independent experiments.

a

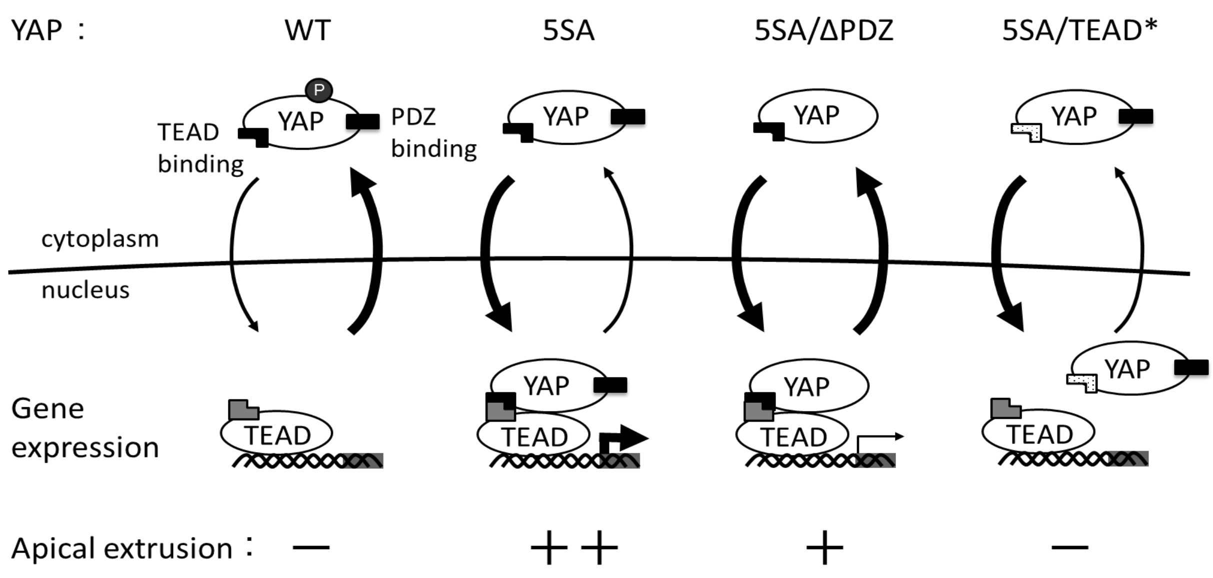
YAP :
WT
5SA
5SA/ΔPDZ
5SA/TEAD*
P
YAP
TEAD
binding
PDZ
binding
cytoplasm
nucleus
Gene
expression
TEAD
Apical extrusion :
—
++
+
—

b

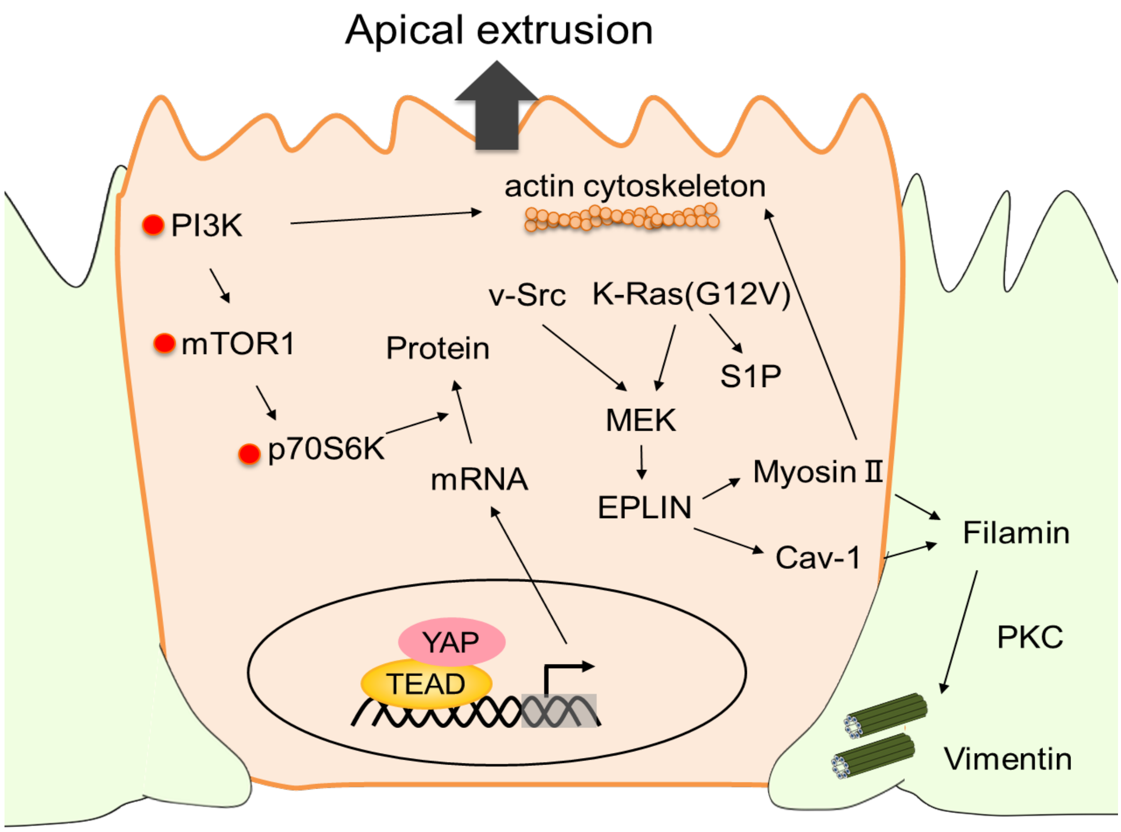
Apical extrusion
actin cytoskeleton
PI3K
mTOR1
p70S6K
Protein
mRNA
v-Src
K-Ras(G12V)
S1P
MEK
EPLIN
Myosin Ⅱ
Cav-1
Filamin
PKC
Vimentin
YAP
TEAD

Supplementary Figure 2. Schematic illustrations of the subcellular localization of YAP mutants and a molecular mechanism for YAP-induced apical extrusion.

(**a**) Schematic illustration of a comparison of the subcellular localization and translocation of YAP, strength of target gene expression, and tendency of cells to undergo apical extrusion, for cells overexpressing WT YAP or the indicated YAP mutants. *, TEAD mutation. Δ, PDZ deletion. (**b**) Schematic illustration of the proposed molecular mechanism of apical extrusion of YAP-, K-Ras (G12V)-, or v-Src-overexpressing cells. Molecules with a red dot are specifically involved in the apical extrusion of YAP (5SA) cells.
